# Integrated printed BDNF-stimulated HUCMSCs-derived exosomes/collagen/chitosan biological scaffolds with 3D printing technology promoted the remodelling of neural networks after traumatic brain injury

**DOI:** 10.1093/rb/rbac085

**Published:** 2022-10-26

**Authors:** Xiaoyin Liu, Jian Zhang, Xu Cheng, Peng Liu, Qingbo Feng, Shan Wang, Yuanyou Li, Haoran Gu, Lin Zhong, Miao Chen, Liangxue Zhou

**Affiliations:** Department of Neurosurgery, West China Hospital, West China Medical School, Sichuan University, Chengdu, Sichuan 610041, China; National Engineering Research Center for Biomaterials, College of Biomedical Engineering, Sichuan University, Chengdu, Sichuan 610064, China; Tianjin Key Laboratory of Neurotrauma Repair, Institute of Traumatic Brain Injury and Neuroscience, Characteristic Medical Center of Chinese People’s Armed Police Force, Tianjin 300162, China; Department of Anesthesiology, West China Hospital, Sichuan University, Chengdu, Sichuan 610064, China; Department of Neurosurgery, West China Hospital, West China Medical School, Sichuan University, Chengdu, Sichuan 610041, China; Department of Liver Surgery & Liver Transplantation, State Key Laboratory of Biotherapy and Cancer Center, West China Hospital, Sichuan University, Chengdu, Sichuan 610041, China; Department of Neurosurgery, West China Hospital, West China Medical School, Sichuan University, Chengdu, Sichuan 610041, China; Department of Neurosurgery, West China Hospital, West China Medical School, Sichuan University, Chengdu, Sichuan 610041, China; The 947th Hospital of Chinese People’s Liberation Army, Xinjiang Uygur Autonomous Region, Kashgar 844000, China; The First Affiliated Hospital of Chengdu Medical College, Chengdu, Sichuan 610500, China; Intensive Care Unit, Traditional Chinese Medicine Hospital of Xinjiang Uyghur Autonomous Region and Affiliated Hospital of Traditional Chinese Medicine of Xinjiang Medical University, Xinjiang Uygur Autonomous Region, Urumqi 830000, China; Department of Neurosurgery, West China Hospital, West China Medical School, Sichuan University, Chengdu, Sichuan 610041, China

**Keywords:** collagen, chitosan, BDNF, exosomes, mesenchymal stem cell, traumatic brain injury

## Abstract

The restoration of nerve dysfunction after traumatic brain injury (TBI) faces huge challenges due to the limited self-regenerative abilities of nerve tissues. *In situ* inductive recovery can be achieved utilizing biological scaffolds combined with endogenous human umbilical cord mesenchymal stem cells (HUCMSCs)-derived exosomes (MExos). In this study, brain-derived neurotrophic factor-stimulated HUCMSCs-derived exosomes (BMExos) were composited with collagen/chitosan by 3D printing technology. 3D-printed collagen/chitosan/BMExos (3D-CC-BMExos) scaffolds have excellent mechanical properties and biocompatibility. Subsequently, *in vivo* experiments showed that 3D-CC-BMExos therapy could improve the recovery of neuromotor function and cognitive function in a TBI model in rats. Consistent with the behavioural recovery, the results of histomorphological tests showed that 3D-CC-BMExos therapy could facilitate the remodelling of neural networks, such as improving the regeneration of nerve fibres, synaptic connections and myelin sheaths, in lesions after TBI.

## Graphical Abstract

**Figure:**
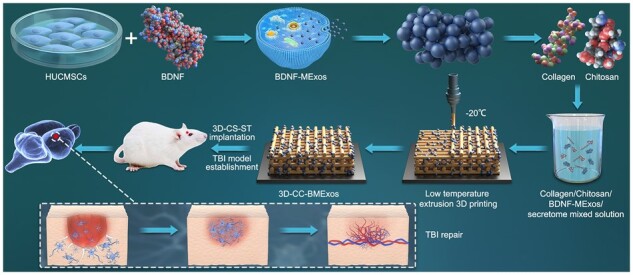

## Introduction

Traumatic brain injury (TBI) is characterized by a high disability rate because of the complexity of injury progression. TBI usually destroys the neuronal interconnections between the brain and the periphery, resulting in temporary or perpetual deprivation of neurological motor abilities [1–7]. A series of secondary injuries, including numerous nerve cell death, inflammatory reaction, necrosis cavum formation and glial scarring, are the main causes of the high disability rate after TBI [8]. Thus, it is a great challenge to prevent secondary injury and improve curative effects after TBI.

The recovery of neurological functions depends on the regeneration and restoration of neural stem cells (NSCs) in the central nervous system. The process of NSCs regeneration and restoration is not only regulated by their own abilities to proliferate and differentiate, but also induced by the external microenvironment. The extracellular matrix, sustentacular cells and cytokines around NSCs constitute a three-dimensional (3D) structure, which is the main component used for neural regeneration [9, 10]. Therefore, the promotion of neural regeneration through improving the local microenvironment in the injury site after TBI to recover neurological defects is currently a hot research field [11]. In recent years, biological tissue engineering has gradually drawn wide attention in the construction of injured neural networks after TBI [12–14]. Biomimetic scaffolds combined with stem cells have been considered a potential therapeutic strategy for remodelling functional tissues to bridge cavity spaces after TBI. Human umbilical cord mesenchymal stem cells (HUCMSCs) therapy has shown positive effects in the repair of neurological function after TBI [15, 16]. However, there are some therapeutic dangers and technical difficulties in the treatment of TBI when using MSCs, such as stem cell transplantation, which easily causes vascular embolism and tumorigenesis [17]. Gradually, methods for the treatment of TBI have evolved from stem cell therapy to cell-free biotherapy, such as exosomes therapy. In particular, HUCMSCs-derived exosomes (MExos) have been shown to have a strong ability to promote the repair and regeneration of damaged neural networks in a TBI rat model [18]. More and more, some studies integrated MSCs-derived exosomes with polypeptide hydrogel matrix, extracellular matrix and other biomaterials in innovative ways, and applied them in the repair of damaged cartilage tissue or myocardial infarction and ultimately observed that exosomes can be released steadily and consistently in the lesion [19, 20]. Xu *et al*. [21] recently revealed that brain-derived neurotrophic factor (BDNF)-induced MSCs-Exos were superior to MSCs-Exos in facilitating cell migration and inhibiting inflammation in a TBI rat model. Moreover, we successfully fabricated collagen/chitosan scaffolds through low temperature extrusion 3D printing technology and demonstrated that 3D-printed collagen/chitosan scaffolds could bridge the injured site and improve the restoration of neuromotor function post spinal cord injury [22].

Based on these conditions, 3D-printed BDNF-stimulated HUCMSCs-derived exosomes (BMExos)/collagen/chitosan/scaffolds scaffolds (3D-CC-BMExos) were prepared through low-temperature extrusion 3D printing as a controlled release system that could accurately deliver exosomes to the lesion in a TBI rat model. The influences of 3D-CC-BMExos scaffolds on the neural regeneration efficacy after TBI were also investigated systematically according to the experimental results.

## Materials and methods

### Extraction and identification of exosomes

All experiments in the study were approved by the Institutional Animal Care and Use Committee of Sichuan University. MSCs derived from the human umbilical cord were purchased from Guangzhou Saliai Stem Cell Co., Ltd and incubated in a complete medium containing 10% Exos-free foetal bovine serum (Gibco Life Technologies, MD, USA) at 37°C and 5% CO_2_. The MSCs cell line were seeded in culture plates and divided into two groups according to the presence or absence of BDNF. Culture plates containing BDNF protein solution (Sigma, St. Louis, USA) (30 ng/ml) were denoted as the BMExos group, and the culture plates without BDNF solution were denoted as the MExos group. The supernatant in both groups was collected after culturing for 48 h. Exosomes were obtained from MSCs supernatant through ultracentrifugation as previously described [23]. MExos and BMExos were assessed for morphology, diameter distribution and specific surface markers (CD9 and CD63) expression through transmission electron microscopy (TEM), NanosizerTM technology and western blotting, respectively. Finally, the MExos and BMExos solutions were sterilized by a 0.22 µm filter (Millipore, Massachusetts, USA) at 4°C.

### Fabrication of scaffolds

The collagen/chitosan mixture was prepared by dissolving 3 g collagen I and 6 g chitosan (Sigma, St. Louis, USA) acetic acid solution (0.05 M, 50 ml), and the mixture was incubated at 4°C overnight. Then, to achieve equilibrium integrating, 0.1 g collagen/chitosan mixed solution was mixed with a solution containing 200 µg of BMExos or 200 µg of MExos, respectively. The two new mixtures were then incubated at 4°C for 24 h. Subsequently, the collagen/chitosan/BMExos mixture and collagen/chitosan/MExos mixture were stirred at 4°C for 12 h and incubated at 4°C overnight again. The 3D-CC-BMExos composite scaffold and 3D-printed collagen/chitosan/MSCs-derived exosomes (3D-CC-MExos) composite scaffold were constructed through 3D printing technology at –20°C. Briefly, two types of mixtures were injected into the printer cartridge of a 3D printer (Regenovo, Hangzhou, China). The parameter values were set in the control panel. After that, the printed scaffolds were transferred into a freezer at –80°C for 12 h followed by vacuum freeze-drying for 48–72 h. The dried scaffolds were further made into a cylinder (2 mm diameter, 2 mm height) by utilizing a hole punch. Similarly, 3D-printed collagen/chitosan scaffolds (3D-CC) were prepared by 3D printing technology under the same conditions. Collagen/chitosan scaffolds (CC) were prepared through a freeze-drying method [24]. Finally, there were four different scaffolds in this study: CC scaffolds, 3D-CC scaffolds, 3D-CC-BMExos scaffolds and 3D-CC-MExos scaffolds. The manufacturing process for composite scaffolds was carried out sterilely at 4°C to maintain the activity of exosomes [22]. Additionally, the morphology of the 3D-CC-BMExos scaffolds was analysed under scanning electron microscope (SEM, Hitachi, Tokyo, Japan). The distribution of PKH26-labelled BExos in the 3D-CC-BMExos scaffolds was observed under a confocal laser scanning microscope (Leica TCS SP5, Germany) [25, 26].

### *In vivo* degradation test of scaffolds

To explore the optimal ratio of collagen and chitosan for the preparation of 3D-CC-BMExos scaffolds, five kinds of 3D-CC-BMExos scaffolds were prepared according to mass ratios (collagen/chitosan) of 5:1, 2:1, 1:2, 1:5 and 1:10. *In vivo* degradation assessment was conducted as previously described [27]. First, we chose 40 male Sprague-Dawley (SD) rats (190 ∼ 200 g) and made three small incisions (diameter of 1 cm) in their backs. The rats were randomly divided into five groups, which were defined as 3D-CC-BMExos group (*n* = 8, collagen/chitosan = 5:1), 3D-CC-BMExos group (*n* = 8, collagen/chitosan = 2:1), 3D-CC-BMExos group (*n* = 8, collagen/chitosan = 1:2), 3D-CC-BMExos group (*n* = 8, collagen/chitosan = 1:5) and 3D-CC-BMExos group (*n* = 8, collagen/chitosan = 1:10). Three composite scaffolds of the same mass ratio were grafted at the injury site of rats (*n* = 3). Then, scaffolds with different mass ratios were removed from the incisions at 1, 2, 3, 4, 5, 6, 7 and 8 weeks (*n* = 8) after surgery to analyse the degradation of composite scaffolds *in vivo*. Briefly, the remaining scaffolds were rinsed with distilled water and placed in a vacuum dryer until the weight was constant. The degradation rate (%) was measured: percent mass remaining = MT/M0 × 100%, where M0 means the initial mass of the 3D-CC-BMExos scaffolds, and MT means the mass of the remaining scaffolds after degradation.

### Physical properties assessment

The water absorption ratio (%) of the scaffolds was also calculated to analyse the physical property. The porosity ratio of the four composite scaffolds (CC, 3D-CC, 3D-CC-BMExos and 3D-CC-MExos) was examined through the ethanol immersion method [8, 28]. Briefly, the initial volume (*V_0_*) and mass (M_0_) of the composite scaffolds were calculated. Then, the scaffolds were immersed in ethanol under negative pressure overnight at 37°C, and the total mass (*M_t_*) was calculated. The porosity ratio (%) was measured using the following formula: porosity ratio (%) = 100% × (*M_t—_M_0_)*/*ρV0*, where ρ is the density of ethanol at 37°C.

### Assessment of the kinetics of BMExos release from composite scaffolds

The samples of 3D-CC-BMExos and CC-BMExos were immersed in PBS at 37°C. The supernatants were collected at 0, 2, 4, 6, 8, 10, 12 and 14 days after incubation, and equal amounts of fresh PBS were then replenished. The BMExos release profile from the 3D-CC-BMExos and CC-BMExos was analysed by a BCA protein assay kit (Thermo Fisher Scientific, USA).

### Exosome uptake

The red fluorescence dye PKH26 (Sigma-Aldrich, USA) was prepared to explore the uptake of BMExos by HUCMSCs or NSCs. Briefly, the BMExos were coincubated with PKH26 at 37°C for 10 min. Then, the PKH26-labelled BMExos were cocultured with HUCMSCs or NSCs for 24 h. Subsequently, the HUCMSCs or NSCs were rinsed with PBS, and the cytoskeleton of HUCMSCs or NSCs was fluorescently stained with FITC phalloidin (Thermo Fisher Scientific, USA) for 1 h. The uptake of PKH26-labelled BMExos by HUCMSCs or NSCs was visualized under a confocal microscope (Leica TCS SP5, Germany).

### Biocompatibility of the composite scaffolds

HUCMSCs at a concentration of 1 × 10^6^/ml were cocultured with 3D-CC-BMExos scaffolds and 3D-CC-MExos scaffolds for 7 days. Then, the morphology and growth of HUCMSCs on the composite scaffolds were observed through phase contrast microscopy (Hitachi, Tokyo, Japan) and haematoxylin and eosin (H&E) staining. MTT assessment was carried out to investigate the cell proliferation on the composite scaffold [29]. Additionally, NSCs were extracted from the hippocampus of SD rats as previously described [25]. NSCs were identified by Nestin immunofluorescence, and the morphology of NSCs was observed with an optical microscope. An MTT cell proliferation assay was also performed to explore the growth of NSCs cocultured with 3D-CC-BMExos scaffolds and 3D-CC-MExos scaffolds. At 7 days after coculture of NSCs and scaffolds, cell adhesion and spreading of NSCs on scaffolds were assessed by using Cytoskeleton staining (Alexa Fluor™ phalloidin, Beyotime, Shanghai, China). After the NSCs were seeded on the surface of the composite scaffolds for 7 days, immunofluorescence staining was performed to explore the differentiation ability of NSCs to form nerve fibres, mature neurons, axons and glial scars. The corresponding primary antibodies were anti-NF (1:200, Abcam, Cambridge, UK), anti-NeuN (1:400, Abcam, Cambridge, UK), anti-GAP43 (1:500, Abcam, Cambridge, UK) and anti-GFAP (1:200, Abcam, Cambridge, UK). To calculate NSCs adhesion rate (%), the 3D-CC-BMExos scaffolds and 3D-CC-MExos scaffolds were placed in 96-well plates. A total of 20 µl of NSCs suspension with a concentration of 5 × 10^6^/ml was seeded on the surface of the composite scaffolds, and then the prepared samples were placed into a 95% air and 5% CO_2_ humidified incubator. The cell adhesion rate of NSCs on the composite scaffolds was analysed 1, 12, 24, 36, 48, 60 and 72 h after coculturing. The formula was as follows: Cell adhesion rate (%) = (number of adherent NSCs/number of seeding NSCs) ×100%.

### Establishment of animal models and transplantation of composite scaffolds

Sixty male SD rats (190–200 g) were prepared and equally divided into four groups: Sham (*n* = 15), TBI (*n* = 15), 3D-CC-MExos (*n* = 15, 3D-CC-MExos transplantation after TBI) and 3D-CC-BMExos (*n* = 15, 3D-CC-BMExos transplantation after TBI). The TBI model was established utilizing a mould. First, the anaesthetized rats were fastened on a stereotactic frame. The skull was exposed, a 5 mm bony window was made by a miniature hand-held cranial drill in the right parietal bone, and the bone flap was 3 mm from the sagittal suture and herringbone suture. After the dura was peeled off, the mould was applied to the brain tissue to form a cylindrical injury cavity (2 mm in diameter and 2 mm in height) in the rat brain tissue of 3D-CC-MExos, 3D-CC-BMExos and TBI groups. The bony window was made without striking in the Sham group. Finally, 3D-CC-MExos and 3D-CC-BMExos were transplanted into the lesion after TBI.

### Neurological function assessment

Morris water maze (MWM) assessment was conducted at 21–27 days after brain injury to explore the spatial learning and memory of rats in each group [30]. Briefly, a cylindrical pool (inner diameter was 180 cm) filled with a mixture of black ink and water was equally divided into four quadrants. Each rat (*n* = 10 in each group) was then forced to swim in the pool to find a hidden platform. The spatial learning study was carried out from Day 21 to 26 and the escape latency was collected. Subsequently, the platform was removed from the pool and the spatial memory study was conducted on Day 27. The quadrant dwell time (swimming time spent in the platform quadrant) and number of site crossings were collected in the spatial memory study. Additionally, modified neurological severity scores (mNSS) were calculated to explore the behavioural function alterations in rats among the four groups at 1, 3, 7, 14, 21 and 28 days after TBI.

### Histological changes

At 2 months after TBI, the brain tissues of the rats among the four groups were fixed in 4% paraformaldehyde after being isolated. All the samples were cut into 5 µm-thick slices using an automatic paraffin slice system. H&E, Bielschowsky’s silver and Nissl staining of brain tissue was carried out to analyse histomorphological alterations and local tissue restoration in the lesion. The quantitative analysis of brain tissue staining was performed utilizing the software ImageJ. Moreover, since TBI can induce the dysfunction of extracranial organs, H&E staining of the liver, kidney, spleen, heart and lung was conducted to investigate their histomorphology changes and the corresponding blood tests were analysed to evaluate their metabolic function after brain injury.

Immunofluorescence staining was also carried out to explore neural restoration after brain injury. Briefly, 5 µm-thick slices were incubated with purified primary antibodies against NF, MBP, NeuN, MAP2 and SYP at 4°C for 12 h. Then, all the samples were incubated with the corresponding secondary antibodies. All antibodies were purchased from Abcam company (UK). Finally, the results of immunofluorescence staining were analysed under a fluorescence microscope (Leica Microsystems, Germany). The relative density of NF-, MBP-, NeuN-, MAP2- and SYP-positive fluorescence staining was calculated using ImageJ software. Additionally, the ultrastructural changes of myelin sheaths and neurons in lesions after TBI were explored by TEM.

### Statistical analysis

All the experimental data were described as mean ± standard deviation (SD). Statistical analysis was carried out utilizing SPSS 15.0. Differences among the multiple groups were conducted by one-way analysis of variance, followed by Student’s t test to conduct comparisons between the two groups. *P *<* *0.05 was considered a significant difference.

## Results

### Characterization of MExos and BDNF-induced MExos

**Figure 1.**
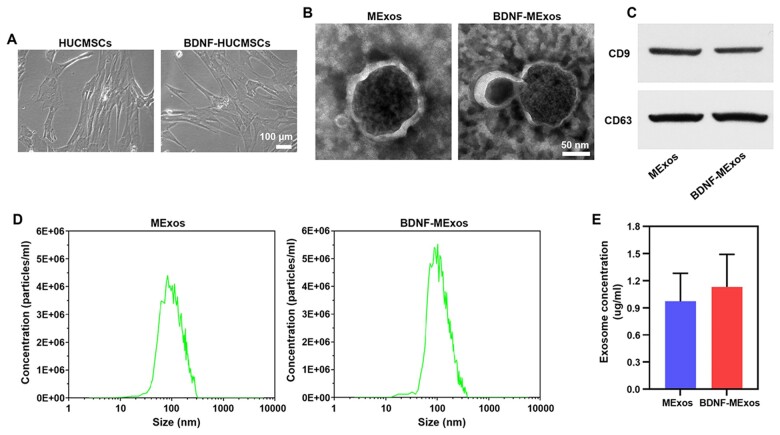
Characterization of Exos derived from HUCMSCs under BDNF conditions and non-BDNF conditions. (**A**) There were no significant alterations in the morphology and growth of HUCMSCs when BDNF was used to stimulate the culture medium of MSCs. (**B**) Morphological characteristics of MExos and BMExos under TEM. (**C**) Exosomal-specific protein markers CD9 and CD63 investigated by western blotting. (**D**) The diameter of MExos and BMExos measured using NanosizerTM technology. (**E**) The concentration of MExos was 0.97 ± 0.31 µg/ml and the concentration of BMExos was 1.13 ± 0.36 µg/ml, no significant differences were found. TEM, transmission electron microscopy.

The morphology and growth of MSCs did not change much when BDNF was added to the culture medium (Fig. 1A). TEM revealed that MExos and BMExos were hollow and oblate microvesicles with a diameter of 30∼150 nm (Fig. 1B). No significant differences were found in micromorphology between MExos and BMExos. The markers CD9 and CD63 of Exos were highly expressed in both MExos and BDNF-induced MExos (Fig. 1C). The peak particle sizes of MExos and BMExos were 110.8 nm and 102.9 nm, respectively (Fig. 1D). The extraction ratio of MExos and BMExos was analysed. The concentrations of MExos and BMExos were 0.97 ± 0.31 µg/ml and 1.13 ± 0.36 µg/ml, respectively, suggesting that the existence of BDNF could slightly increase the productivity of exosomes in the culture medium, although no significant differences were found between the two groups (Fig. 1E).

### Properties of composite scaffolds

Considering that the biological composite scaffold will be applied in clinical in the future, so we chose HUCMSCs to extract exosomes. Additionally, we referred to the experimental methods in many literatures when we designed the experiment. And all of the literatures described that the HUCMSCs-derived exosomes can be utilized to treat the SD rats with different diseases [31, 32]. Therefore, we used HUCMSCs to extract exosomes and prepare 3D-CC-BMExos for animal experiments.

**Figure 2.**
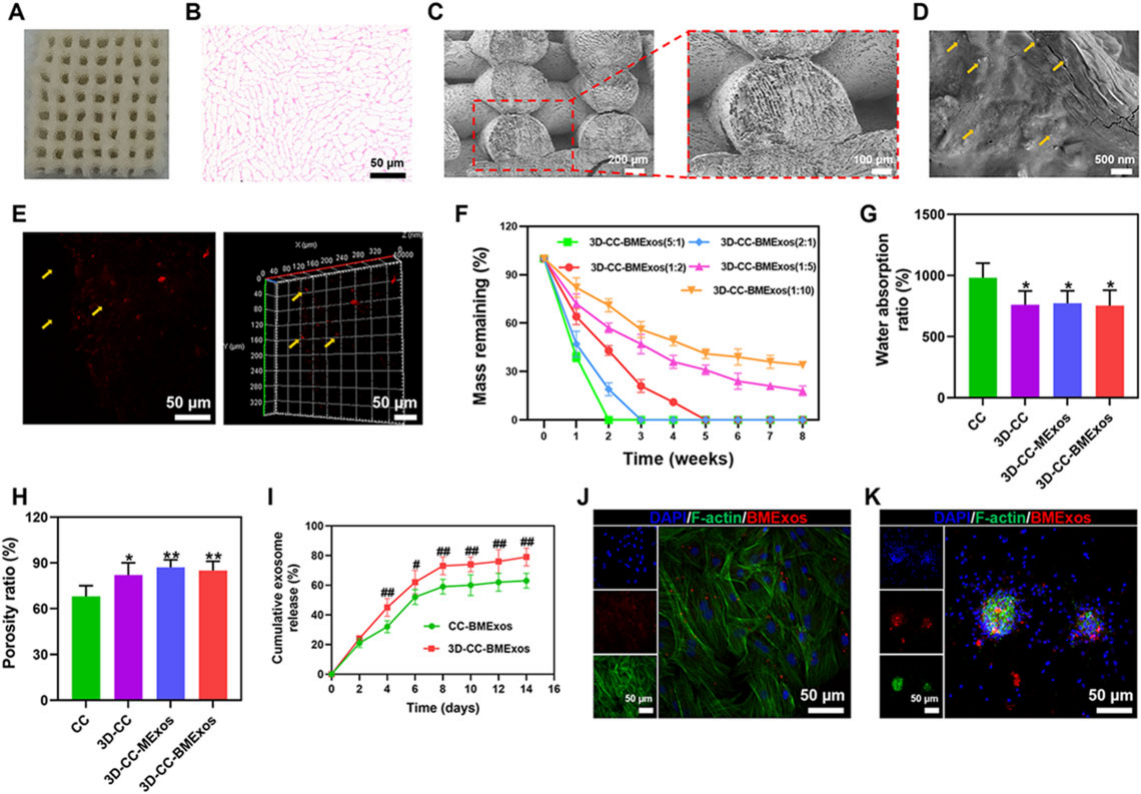
Characteristics of composite scaffolds. (**A**–**D**) Representative images of 3D-CC-BMExos under general observation (A), H&E staining (**B**) and SEM (**C** and **D**). The pores of 3D-CC-BMExos were well connected to each other and the BMExos were evenly distributed in the 3D-CC-BMExos scaffold (D). (**E**) Representative 3D immunofluorescence images showed the distribution of BMExos in 3D-CC-BMExos. (**F**) Degradation rate of 3D-CC-BMExos prepared with different collagen/chitosan mass ratios at 2, 4, 6 and 8 weeks after transplantation. (**G** and **H**) Compared with CC scaffolds, 3D-printed composite scaffolds showed lower water absorption ratio and higher porosity ratio. (**I**) Cumulative release profile of BMExos from the CC-BMExos and 3D-CC-BMExos within 14 days. (**J** and **K**) Representative images of F-actin/PKH26-labelled BMExos immunofluorescence staining in HUCMSCs (J) and NSCs (K). All data were expressed as mean ± SD; *P <* *0.05, **P <* *0.01 vs CC; ^#^*P *<* *0.05, ^##^*P *<* *0.01 vs CC-BMExos; SEM, scanning electron microscope.

We found that the pores of 3D-CC-BMExos were evenly distributed and well connected to each other under the general observation, SEM and H&E staining (Fig. 2A–C). The excellent porous microstructure is favourable for cell growth, migration and adhesion [33]. The results of SEM (Fig. 2D) and immunofluorescence images (Fig. 2E) also demonstrated that BMExos were evenly distributed in the 3D-CC-BMExos, suggesting that BMExos were successfully integrated with the 3D-CC during the 3D printing process. The composite scaffolds prepared by different collagen/chitosan mass ratios had different degradability. Figure 2F showed that 3D-CC-BMExos scaffolds prepared by two of the five mass ratios (collagen/chitosan 1:5 and 1:10) were not completely degraded at 8 weeks after transplantation. The composite scaffolds with mass ratios of 5:1 and 2:1 (collagen/chitosan) were thoroughly degraded at 4 weeks after implantation, and the composite scaffolds were thoroughly degraded at 5 weeks when the collagen/chitosan mass ratio was 1:2 (Fig. 2F). These results showed that the degradation ability of composite scaffolds was accelerated with increasing collagen proportion. Based on the degradation characteristics of 3D-CC-BMExos with different mass ratios of collagen/chitosan, 3D-CC-BMExos with a mass ratio of 1:2 (collagen/chitosan) were used for subsequent experiments because *in vivo* experiments were performed at 2 months after TBI. Moreover, the composite scaffolds including 3D-CC, 3D-CC-MExos and 3D-CC-BMExos, prepared through 3D printing technology exhibited excellent physical properties. Compared with CC, 3D-printed composite scaffolds had lower water absorption ratio (Fig. 2G) (*P *<* *0.05) and higher porosity ratio (Fig. 2H) (*P *<* *0.05). Lower water absorption prevented Exos loss and controlled the release of Exos from the composite scaffolds. The good porosity ratio was beneficial for transferring the nutrient solution and forming new tissue. Figure 2I showed the release profile of BMExos from CC-BMExos scaffolds and 3D-CC-BMExos scaffolds. From Day 4, the BMExos released from 3D-CC-BMExos were significantly higher than those released from CC-BMExos (Fig. 2I) (*P *<* *0.05), suggesting that BMExos in 3D-CC-BMExos scaffolds could be released more completely to generate biological activity in the repair of damaged tissue. Additionally, PKH26-labelled BMExos were observed in the cytoplasm of HUCMSCs (Fig. 2J) and NSCs (Fig. 2K), indicating successful *in vitro* endocytosis of BMExos released from 3D-CC-BMExos.

### Biocompatibility of composite scaffolds

**Figure 3.**
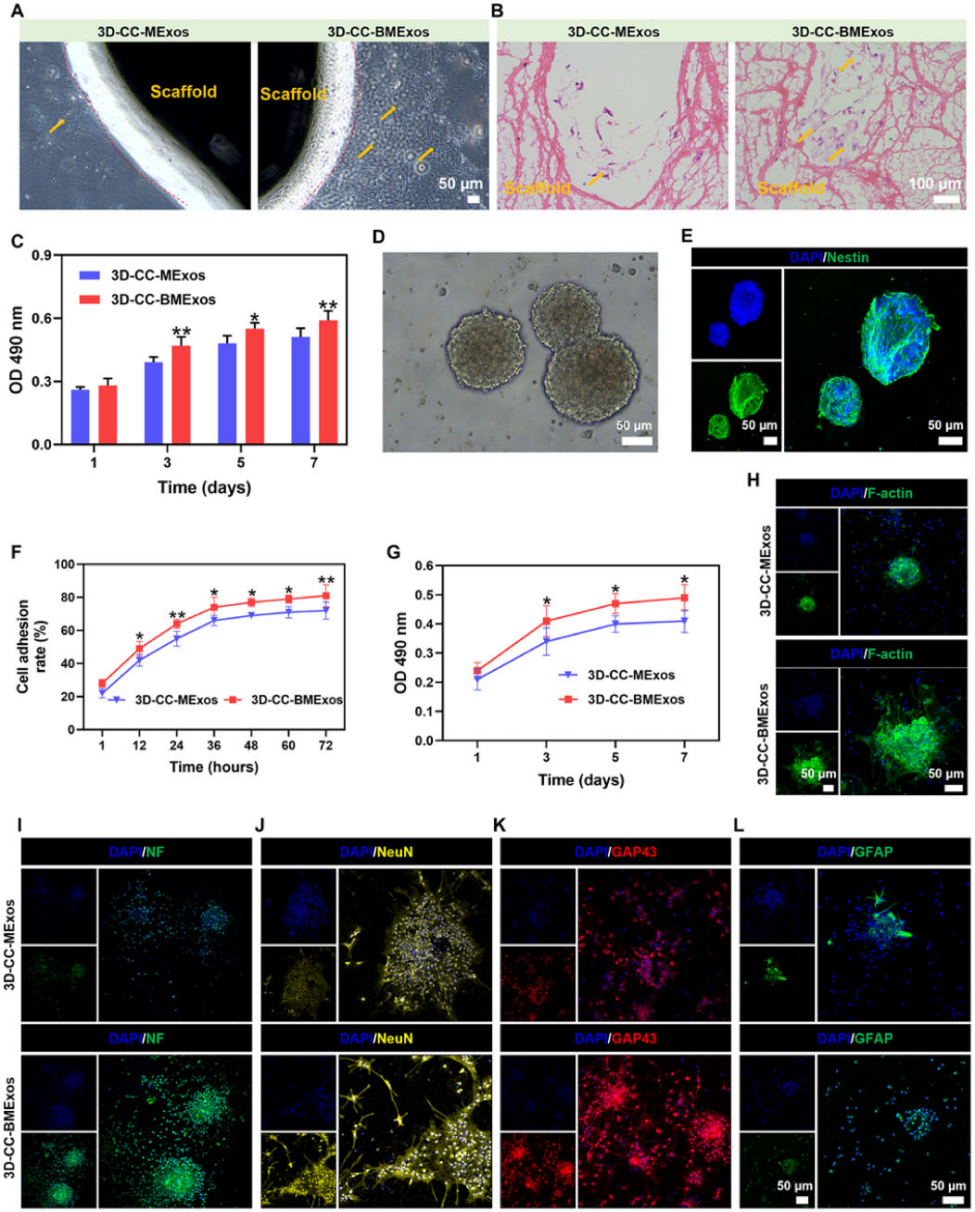
Biocompatibility test of scaffolds. (**A** and **B**) Representative images of HUCMSCs cocultured with 3D-CC-MExos or 3D-CC-BMExos under phase contrast microscopy (A) and H&E staining (B). (**C**) MTT assay of HUCMSCs cocultured with 3D-CC-MExos and 3D-CC-BMExos. (**D**) NSCs were spherical under an optical microscope. (**E**) Representative immunofluorescence images of NSCs stained with a nestin antibody (green). (**F**) Cell adhesion rates of NSCs at different time points after coculture of NSCs with scaffolds. (**G**) MTT assay of NSCs cocultured with 3D-CC-MExos and 3D-CC-BMExos. (**H**–**L**) Representative image of F-actin (H), NF (I), NeuN (J), GAP42 (K) and GFAP (L) immunofluorescence staining at 7 days after coculture. All data were expressed as mean ± SD; *P <* *0.05, **P <* *0.01 vs 3D-CC-MExos.

We observed that HUCMSCs grew well on both 3D-CC-MExos and 3D-CC-BMExos under a phase contrast microscope (Fig. 3A). H&E staining of composite scaffolds after 7 days of coculture also confirmed that the composite scaffolds were suitable for cell growth (Fig. 3B). However, the density of HUCMSCs on 3D-CC-BMExos was obviously larger than that on 3D-CC-MExos (Fig. 3A and B), indicating that 3D-CC-BMExos were more suitable for cell proliferation. Similar results were observed from the MTT assay of HUCMSCs (Fig. 3C) (*P *<* *0.05). Since NSCs play important roles in the remodelling neural network after TBI, we further detected the biocompatibility between NSCs and 3D-printed composite scaffolds. First, we observed and determined the morphology of NSCs under an optical microscope (Fig. 3D) and immunofluorescence microscope (Fig. 3E). With increasing coculture time, the cell adhesion rate of NSCs on 3D-CC-MExos and 3D-CC-BMExos increased. There was a significant difference in the cell adhesion rate between the two groups after 12 hours of coculture of NSCs and scaffolds (Fig. 3F) (*P *<* *0.05). Moreover, an MTT assay of NSCs was also conducted to detect NSCs proliferation on the 3D-printed scaffolds. The OD value in the 3D-CC-BMExos group was higher than that in the 3D-CC-MExos group (Fig. 3G) (*P *<* *0.05), suggesting that the cell viability of NSCs in the 3D-CC-BMExos scaffolds was better. The effects of 3D-CC-BMExos scaffolds on neural differentiation were also detected by immunofluorescence after the NSCs were cocultured with composite scaffolds for 7 days. Compared with the 3D-CC-MExos group, the positive areas of F-actin, NF, NeuN and GAP43 were higher in the 3D-CC-BMExos group (Fig. 3H–K). These results suggested that 3D-CC-BMExos have a stronger ability to promote NSCs differentiation into nerve fibres, neurons and axons. The GFAP-positive area was significantly lower in the 3D-CC-BMExos group than in the 3D-CC-MExos group, revealing that the 3D-CC-BMExos scaffolds did not facilitate the differentiation of NSCs into astrocytes (Fig. 3L). This is beneficial in preventing the formation of glial scarring at the lesion after TBI.

### 3D-Cc-BMExos therapy improved the recovery of cognitive function and sensorimotor function

**Figure 4.**
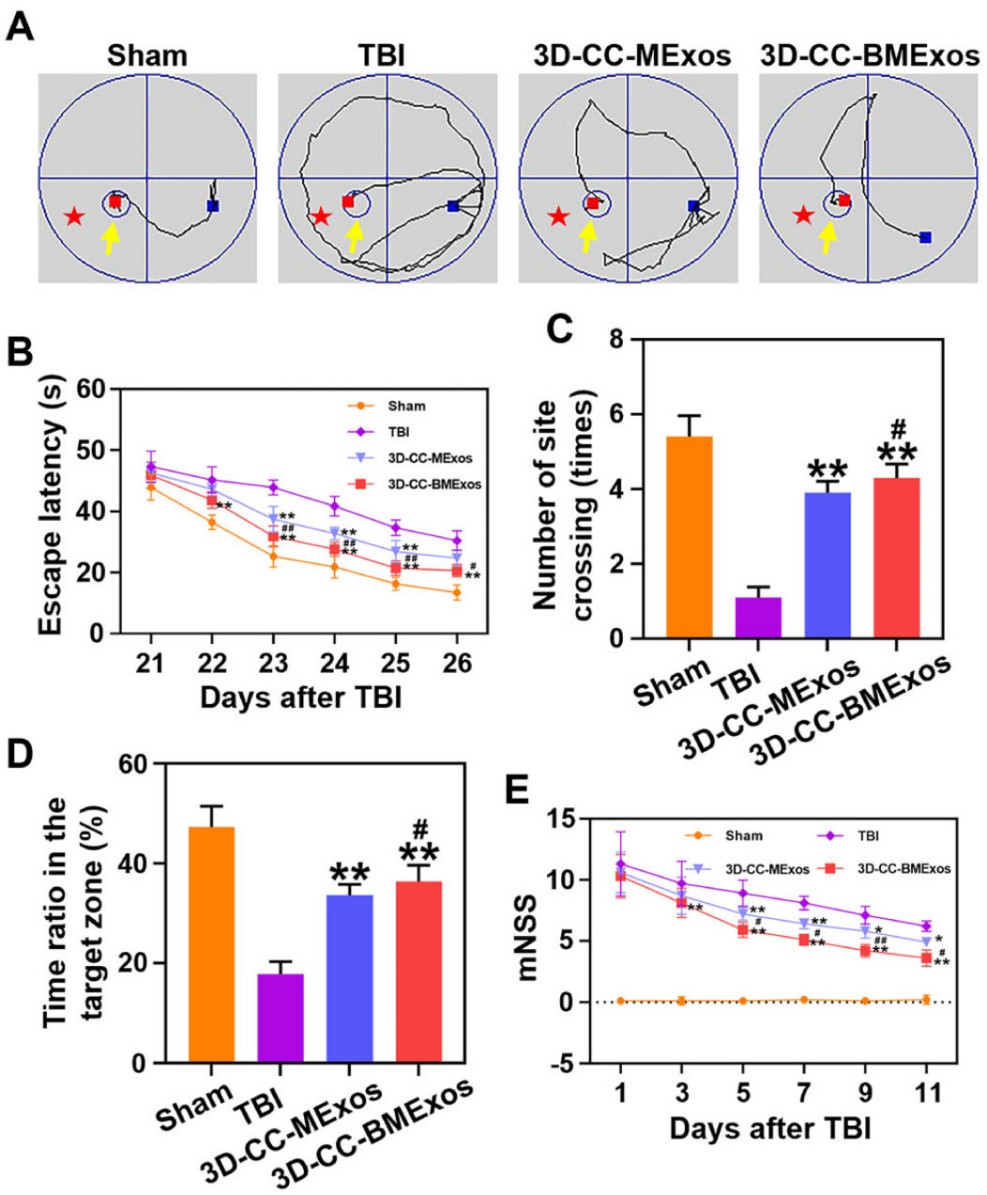
The recovery of cognitive function and sensorimotor function among the four groups. (**A**) Representative images of the search route in the spatial learning stage. The yellow arrow represents the platform, and the zone labelled by the red five-pointed star is the target zone. (**B**) The analysis of escape latency in the spatial learning stage. (**C** and **D**) Analysis of the number of site crossings (C) and the time ratio in the target zone (D) in the spatial memory stage. (**E**) mNSS assessment at different time points after TBI. All data were collected as mean ± SD; *P <* *0.05, **P <* *0.01 vs TBI group; ^#^*P *<* *0.05, ^##^*P *<* *0.01 vs 3D-CC-MExos group.

MWM assessment was performed to detect the recovery of cognitive function. The spatial learning and memory ability of rats in the 3D-CC-BMExos group and 3D-CC-MExos group were improved after composite scaffolds transplantation (Fig. 4A–D). The rats in the TBI group showed an obvious cognitive defect compared to the rats in the Sham group (Fig. 4A). Administration of 3D-CC-BMExos therapy could significantly improve the recovery of cognitive functions by decreasing escape latency (Fig. 4B) and increasing the number of site crossings (Fig. 4C) and the time ratio in the target zone (Fig. 4D) compared to the rats in the 3D-CC-MExos group (*P *<* *0.05). These results indicate that transplantation of 3D-CC-BMExos in the lesion after TBI had protective effects on TBI-induced cognitive dysfunction. mNSS was conducted to analyse the recovery of motor and sensory functions (Fig. 4E). No significant differences were found in mNSS scores among the 3D-CC-BMExos group, 3D-CC-MExos group and TBI group at Day 1 after TBI. Subsequently, the 3D-CC-BMExos group and 3D-CC-MExos group exhibited an obvious downwards trend from Days 5 to 28 compared with the TBI group (*P *<* *0.05). In particular, the mNSS score of the 3D-CC-BMExos group was significantly lower than that of the 3D-CC-MExos group from Day 5 (*P *<* *0.05), indicating that 3D-CC-BMExos therapy could further improve the recovery of sensorimotor function after TBI.

### 3D-Cc-BMExos therapy improved the remodelling of neural networks

**Figure 5.**
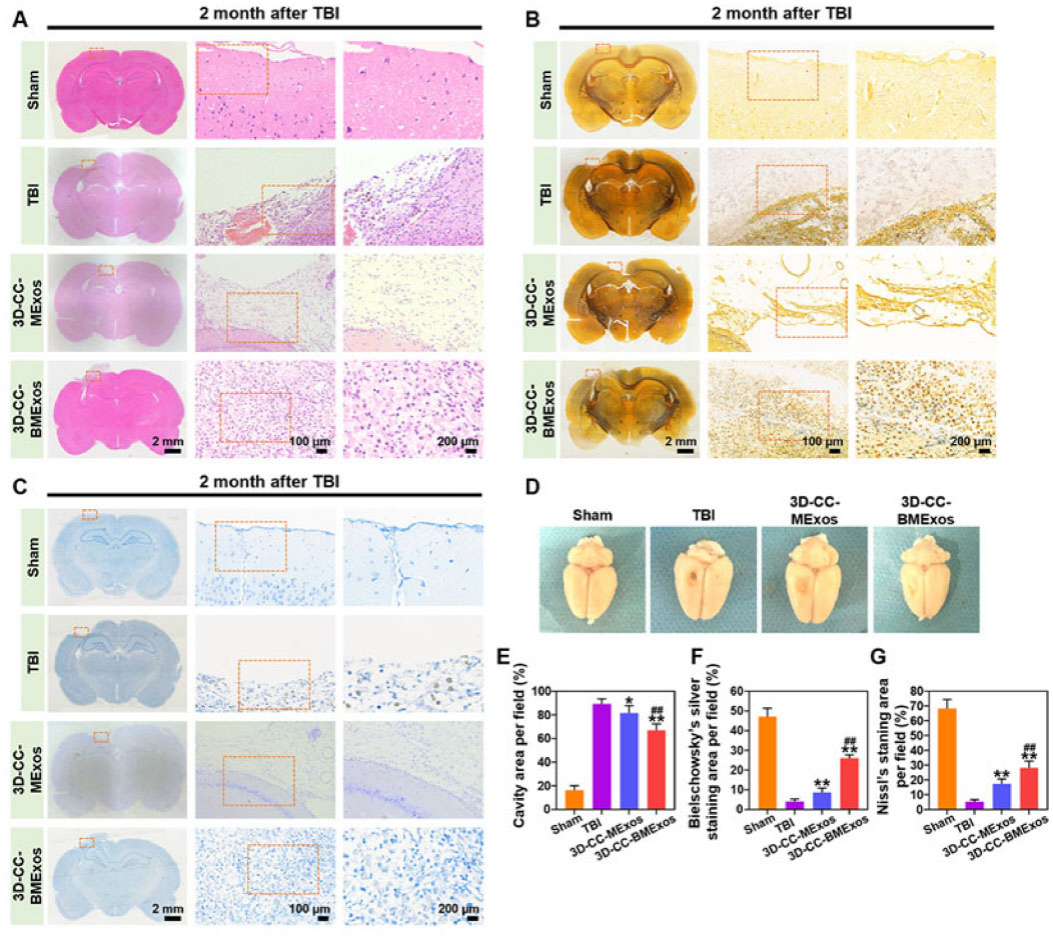
3D-CC-BMExos therapy promoted histomorphological recovery at the injury site after TBI. (**A**) Representative H&E-stained images of brain slices among the four groups showing the differences in histomorphology at 2 months after TBI. (**B**) Representative Bielschowsky’s silver staining images of brain slices among the four groups showing the differences in nerve fibre alterations at 2 months after TBI. (**C**) Representative Nissl staining images of brain slices among the four groups showing the differences in the numbers of neuronal cell bodies at 2 months after TBI. (**D**) General observation of cavity after TBI among the four groups. (**E**) Quantitative measurement of cavity area per field at the defect site. (**F**) Quantitative measurement of bielschowsky’s silver staining area per field at the defect site. (**G**) Quantitative measurement of nissl staining area per field at the defect site. All data were expressed as mean ± SD; *P <* *0.05, **P <* *0.01 vs TBI group; ^##^*P *<* *0.01 vs 3D-CC-MExos group.

The results of H&E staining of brain tissue showed that there was severe damage and large cavities at the injury site in the TBI group (Fig. 5A and E). Interestingly, the injury cavities are obviously larger than 2 mm in diameter and 2 mm in height. This is because that the regular cavity at the injury site of brain tissue only lasts for a short time after the TBI model of rats is established with a mould. However, it has been well known that liquefaction necrosis will occur around the lesion and lead to changes in the size of the lesion with the prolonging of time after brain injury. Therefore, the diameter and height of the injured cavity were significantly larger than 2 mm, and the lesions showed irregular shape. The cavity area was significantly smaller in the 3D-CC-BMExos and 3D-CC-MExos groups. In particular, compared with the 3D-CC-MExos group, more regenerated tissue was observed in the cavity in the 3D-CC-BMExos group (*P *<* *0.01). The results of Bielschowsky’s silver staining reflected nerve fibre alterations in the cavity. As shown in Fig. 5B, there were abundant nerve fibres in the cavity in the 3D-CC-BMExos and 3D-CC-MExos groups, while few nerve fibres were found in the TBI group. Quantitative analysis (Fig. 5F) revealed that the area of nerve fibres in the 3D-CC-BMExos and 3D-CC-MExos groups was significantly larger than that in the TBI group (*P *<* *0.01). Compared with the 3D-CC-MExos group, the distribution of nerve fibres was more widespread in the 3D-CC-BMExos group (*P *<* *0.01). Similar alterations were observed in Nissl staining of brain tissue (Fig. 5C and G), and Nissl bodies were more widely distributed in the 3D-CC-BMExos group than in the 3D-CC-MExos (*P *<* *0.01) and TBI groups (*P *<* *0.01). The number of Nissl bodies in the 3D-CC-MExos group was significantly higher than that in the TBI group (*P *<* *0.01). Moreover, a general view of the brain tissue (Fig. 5D) also showed that the 3D-CC-BMExos group had a smaller cavity, indicating that 3D-CC-BMExos therapy had a positive significance in repairing damaged brain tissue after TBI.

**Figure 6.**
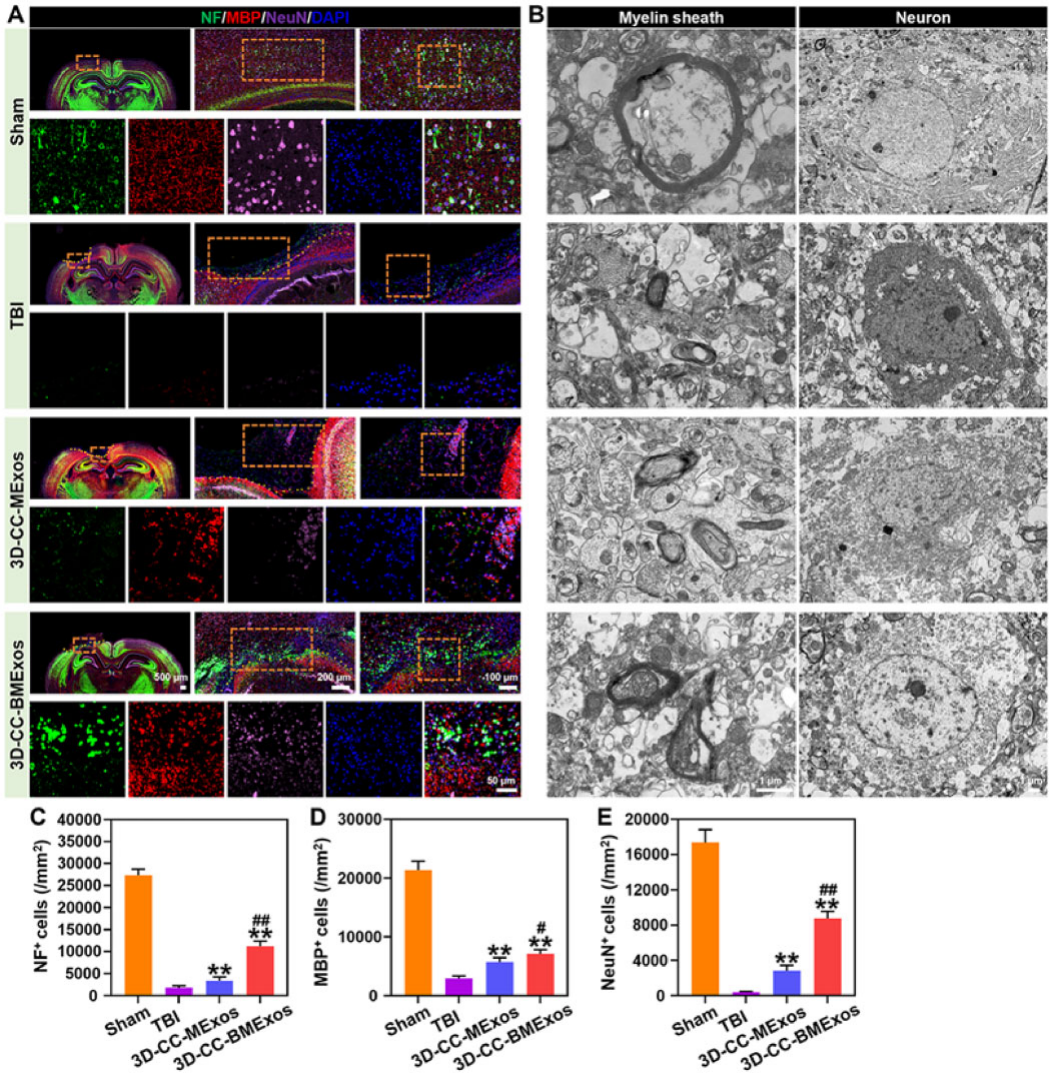
The recovery of nerve fibres, myelin sheaths and neurons at the injury site of TBI. (**A**) Representative immunofluorescence images of nerve fibres, myelin sheaths and neurons in the injury site. (**B**) Representative TEM images of myelin sheaths and neurons. (**C–E**) The quantification of NF^+^ cells (C), MBP^+^ cells (D) and NeuN^+^ cells (E) in the injury site among the four groups. All data were expressed as mean ± SD; **P <* *0.01 vs TBI group; ^#^*P *<* *0.05, ^##^*P *<* *0.01 vs 3D-CC-MExos group; TEM, transmission electron microscope.

**Figure 7.**
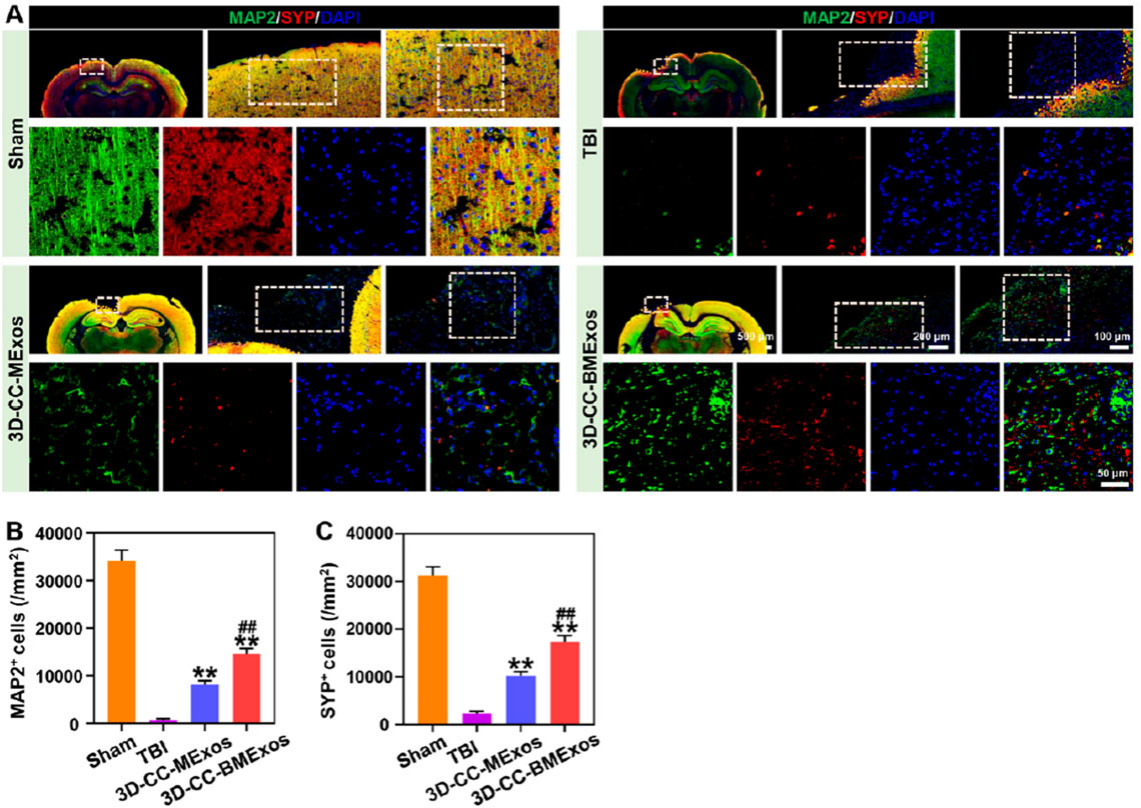
The recovery of synapses and axons at the injury site of TBI. (**A**) Representative immunofluorescence images of axons and synapses in the injury site. (**B** and **C**) Quantification of the MAP2^+^ cells (B) and SYP^+^ cells (C) in the injury site among the four groups. All data were expressed as mean ± SD; **P <* *0.01 vs TBI group; ^##^*P *<* *0.01 vs 3D-CC-MExos group.

Immunofluorescence labelling with specific antibodies against NF/MBP/NeuN and MAP2/SYP was conducted to further detect whether 3D-CC-BMExos promoted the remodelling of neural networks at the lesion after TBI. The results of immunofluorescence staining revealed that the lesions covered by NF/MBP/NeuN (Fig. 6A) and MAP2/SYP (Fig. 7A) as well as their protein levels were highest in the 3D-CC-BMExos group (*P *<* *0.01, vs TBI group; *P *<* *0.05, vs 3D-CC-MExos group). Moreover, the number of MBP/NeuN/NF (Fig. 6C–E) and SYP/MAP2 (Fig. 7B and C) positive cells was larger in the 3D-CC-MExos group than in the TBI group (*P *<* *0.01). These results revealed that transplantation of 3D-CC-BMExos in the injury site after TBI could significantly accelerate the restoration of myelin sheaths, neurons, nerve fibres, synapses and axons.

For the TEM assay, the structure of the myelin sheath and neurons in the Sham group was intact with a large diameter, and no obvious deformation was observed (Fig. 6B). However, the other three groups showed a significant alteration in the structure of myelin sheath and neurons (Fig. 6B). Compared with the TBI group, the myelin sheath in the 3D-CC-BMExos and 3D-CC-MExos groups was thicker and had a larger diameter, and the membrane structure of neurons was more complete (Fig. 6B). Additionally, compared with the 3D-CC-MExos group, the diameter of the myelin sheath in the 3D-CC-BMExos group was larger, and the morphology and membrane structure of neurons were more complete (Fig. 6B).

### Toxicity assessment of composite scaffolds degradation products *in vivo*

**Figure 8.**
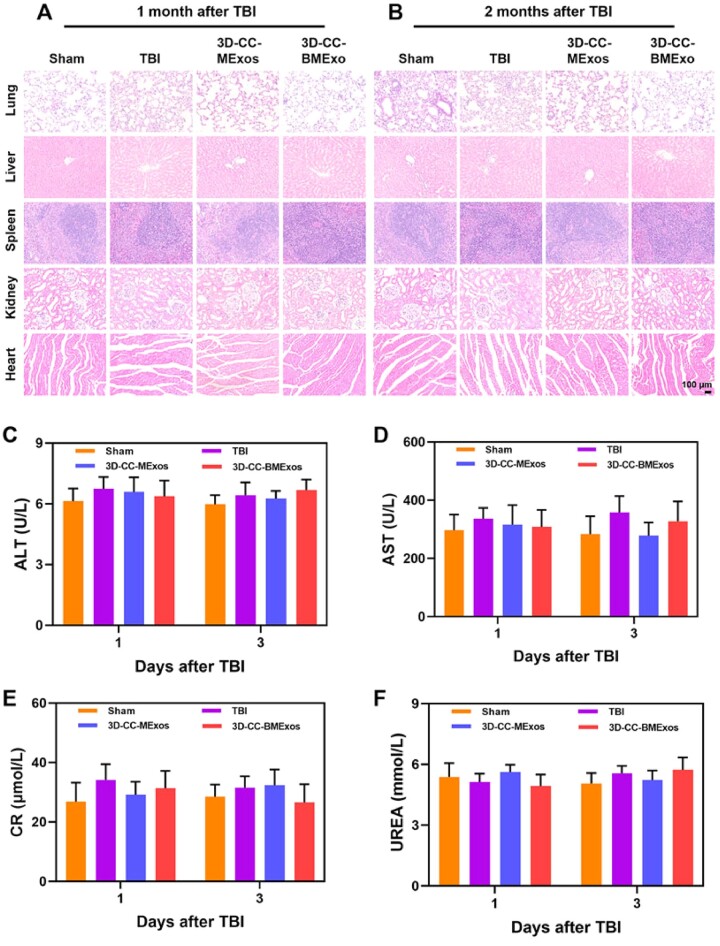
*In vivo* toxicity of transplanted composite scaffolds. (**A** and **B**) H&E staining of the lung, liver, spleen, kidney and heart at 1 month and 2 months after TBI among the four groups. (**C**–**F**) There were no significant differences in lab blood tests of ALT, AST, CR and UREA among the four groups. ALT, alanine aminotransferase; AST, aspartate aminotransferase; CR, serum creatinine; UREA, blood urea nitrogen.

Compared with the Sham group, no significant pathological abnormalities were found in major organs (such as the lung, liver, spleen, kidney and heart) at 1 month and 2 months after TBI in the other three groups (Fig. 8A and B). The corresponding lab blood tests showed that the functional indicators of the liver and kidney (Fig. 8C–F), such as alanine aminotransferase, aspartate aminotransferase, serum creatinine and blood urea nitrogen, among the four groups were at the same level. These results indicated that 3D-CC-BMExos therapy did not induce systemic toxicity *in vivo*.

## Discussion

MExos have been suggested to be a potential strategy in the treatment of TBI [23]. In particular, MExos induced by BDNF showed a better ability to promote the recovery of neural networks in TBI rats by regulating miR-216a-5p [21]. In this study, we mixed BMExos and MExos with collagen/chitosan to prepare 3D-CC-BMExos and 3D-CC-MExos by 3D printing technology, and transplanted the 3D-printed scaffolds directly into the injury site of TBI rats to investigate their therapeutic effect. Finally, behavioural and histomorphological tests showed that 3D-CC-BMExos therapy could significantly improve the recovery of neurological function in TBI rats.

It is well known that composite scaffolds prepared by collagen and chitosan have favourable biocompatibility and mechanical properties, which can provide essential cell–cell and cell–microenvironment interactions to promote the repair of damaged tissue [34]. Collagen has the advantages of good biocompatibility and low immunogenicity, but it also has the disadvantage of easy degradability *in vivo* [35, 36]. Chitosan has been shown to be effective in stimulating haemostasis, accelerating tissue regeneration and enhancing the activity of cell proliferation. Chitosan also acts as an antimicrobial agent to prevent bacterial and fungal infections at the injure site [37, 38]. Moreover, chitosan has low hydrophilicity and poor degradability, and can generate positive charges to bind with the amino and carboxyl hydrophilic groups of collagen molecules through electrostatic interactions [39, 40]. Therefore, the binding of chitosan can effectively decrease the degradation rate of collagen. Multiple studies have shown that both collagen and chitosan play important roles in neural network reconstruction after central nervous system injury [41, 42]. Therefore, collagen and chitosan were considered as the essential materials to prepare biological scaffolds in this study. Additionally, with the help of 3D printing technology, the shape, size, porosity ratio and morphology of the composite scaffolds can be better adjusted for *in vivo* transplantation [43]. Figure 2G shows that 3D-printed scaffolds have suitable water absorption ratio, which can prevent the loss of body fluids and nutrients and is conducive for cell growth and tissue regeneration. It should be pointed out that the mixture of exosomes, collagen and chitosan were 3D-printed at a low temperature (4°C), which is beneficial to maintain the bioactivity of exosomes. In our study, we found that the composite scaffold printed under a low temperature could well control the release of exosomes (Fig. 2J), and the exosomes released by the scaffold could be successfully swallowed by stem cells (Fig. 2K and L) and promoted the differentiation of NSCs into different nerve cells. These results further demonstrated that the 3D printing process conducted at a low temperature could minimize the loss of bioactivity of exosomes. More importantly, the cumulative exosome release of 3D-CC-BMExos scaffolds was significantly higher than that of CC-BMExos scaffolds, indicating that the ability of collagen/chitosan to immobilize BMExos was enhanced through 3D printing technology. The release of BMExos can be controlled by the porosity and degradation rate of the scaffolds [25]. The porosity ratio of 3D-CC-BMExos was higher than that of CC scaffolds, which might be the reason why 3D-CC-BMExos released exosomes for a longer time and in a larger amount than CC-BMExos. In addition, the results of SEM showed that the interior of 3D-CC-MExos was a porous network structure, and BMExos adhered to the inner surface of 3D-CC-MExos. Moreover, the results of immunofluorescence staining also demonstrated the internal distribution of BMExos in 3D-CC-MExos. All in all, these results could show that BMExos have been successfully loaded onto 3D-CC-MExos [44]. The 3D printing technology can make the exosomes evenly mixed and adhered to the scaffold, so the exosomes released by 3D-CC-BMExos are more durable and the total amount released was higher than that of CC-BMExos.

Extracellular vesicles contain many exosomes, microvesicles and apoptotic bodies. Exosomes extracted by different methods are slightly different in particle size and purity. However, exosomes are generally not more than 200 nm in size [45], while microvesicles can be up to 500 nm or even more than 1000 nm in size. Even though it will spend more time extracting exosomes by ultracentrifugation, it is still the best way to obtain the exosomes of high purity [46]. Therefore, ultracentrifugation was used to extract exosomes in our study. There are various proteins, lipids, RNA, mRNAs and miRNAs in exosomes that are involved in promoting the remodelling of neural network after TBI by inhibiting cell apoptosis and regulating the immune system [47, 48]. Additionally, the biological functions of exosomes derived from MSCs may be different in different organs, suggesting that adverse reactions may be observed in some other organs when exosomes are used to treat TBI [18, 49]. Therefore, it is necessary to enhance the specificity of exosomes in the clinic to effectively improve the exosome concentration in the central nervous system and prevent adverse effects on other organs.

In the current study, the fabricated 3D-CC-BMExos scaffolds significantly promoted the regeneration efficacy of nervous tissue defects in a model of TBI rats. Future studies should be conducted to explore the tissue repair processes to provide favourable thoughts on scaffold design. For instance, tracing exosomes in the lesion can be performed to observe the penetration and distribution of exosomes. Additionally, it is necessary to study the cell classification associated with neural regeneration in the healing process to guide the repair of normal tissue by regulating the properties of the transplanted biological scaffolds.

## Conclusions

In this study, we put proposed a potential therapeutic strategy for TBI based on BDNF-stimulated HUCMSCs-derived exosomes and 3D-printed collagen/chitosan scaffolds. The high porosity ratio and excellent biocompatibility of 3D-CC-BMExos scaffolds could provide a favourable microenvironment for cell proliferation and differentiation *in vitro* and *in vivo*. In conclusion, 3D-CC-BMExos therapy has positive effects on improving neural regeneration after TBI and possesses greater potential for use in the clinic in the future.

## Funding

This work was supported by the National Major Scientific and Technological Special Project for Significant New Drugs Development (2015ZX09102010).

*Conflicts of interest statement*. None declared.
